# DeepCOMO: from structure-activity relationship diagnostics to generative molecular design using the compound optimization monitor methodology

**DOI:** 10.1007/s10822-020-00349-3

**Published:** 2020-10-05

**Authors:** Dimitar Yonchev, Jürgen Bajorath

**Affiliations:** Department of Life Science Informatics, B-IT, LIMES Program Unit Chemical Biology and Medicinal Chemistry, Rheinische Friedrich-Wilhelms-Universität, Endenicher Allee 19c, 53115 Bonn, Germany

**Keywords:** Analog series, Lead optimization, Chemical saturation, SAR progression, Activity prediction, Generative deep learning

## Abstract

The compound optimization monitor (COMO) approach was originally developed as a diagnostic approach to aid in evaluating development stages of analog series and progress made during lead optimization. COMO uses virtual analog populations for the assessment of chemical saturation of analog series and has been further developed to bridge between optimization diagnostics and compound design. Herein, we discuss key methodological features of COMO in its scientific context and present a deep learning extension of COMO for generative molecular design, leading to the introduction of DeepCOMO. Applications on exemplary analog series are reported to illustrate the entire DeepCOMO repertoire, ranging from chemical saturation and structure–activity relationship progression diagnostics to the evaluation of different analog design strategies and prioritization of virtual candidates for optimization efforts, taking into account the development stage of individual analog series.

## Introduction

The intuition- and experience-driven process of hit-to-lead and lead optimization (LO) presents key challenges for medicinal chemistry. If successful, it ranges from the initial demonstration of sustainable structure–activity relationships (SARs) of selected active compounds and the iterative generation of many analogs to the final stages of confirming pre-clinical candidate status of optimized compound(s). To this date, the LO process is difficult, if not impossible to rationalize. Work on analog series (ASs) continues until multi-property optimization criteria are met or insurmountable roadblocks are hit. This typically is far from being a black-and-white scenario. Partly unclear SAR responses or rather subtle differences between desirable and undesirable compound properties often propagate through optimization efforts until they amplify and result in large-magnitude problems. At such stages, when much work has already been spent on the long road to candidate compounds, it is often difficult to call it a day and discontinue work on advanced series. As a matter of fact, answering the question when sufficient numbers of analogs might have been generated and further progress would be unlikely to expect is at least as critical in the practice of medicinal chemistry as making meaningful initial decisions which compounds or series to advance or not. In light of these caveats looming over optimization efforts, it is self-evident that any approaches providing decision support during LO are more than welcome. However, the problems associated with empirical optimization are conceptually difficult to tackle. Currently, only a limited number of computational approaches are available that are capable of supporting LO efforts. This is the scientific context in which the Compound Optimization MOnitor (COMO) methodology evolved. One of the roots of COMO was the development of a scoring scheme to evaluate chemical saturation of compound series on the basis of biological screening data [1, 2]. Modifying and extending this scoring scheme and combining it with the assessment of SAR progression then gave rise to the introduction of the COMO approach [3–5], which was originally designed as a diagnostic. On the basis of COMO scoring, ASs can be assigned to different development stages. An integral feature of the COMO approach is the use of virtual analog (VA) populations to aid in the assessment of chemical saturation and SAR progression. By default, these VAs also represent potential candidate compounds for LO. Thus, although COMO was originally devised as a diagnostic/descriptive tool it also had the intrinsic potential to bridge between LO analysis and compound design. Accordingly, different analog design strategies and activity prediction approaches have been implemented in COMO to design and prioritize VAs [5, 6].

Herein, we report a methodological extension of COMO’s analog design strategies through deep learning and generative modeling using recurrent neural networks (RNNs). Accordingly, the combined diagnostic scoring and extended analog design approach is termed DeepCOMO. In addition, we discuss current computational approaches having the potential to support different stages of LO efforts. In this context, we also describe key components of the DeepCOMO methodology. Furthermore, we present an application of DeepCOMO on two exemplary ASs, illustrating its entire analysis and design spectrum, as it has evolved since its inception [6]. Here, emphasis is put on the compound design aspect applying the DeepCOMO framework.

The subsequent sections are organized as follows. First, we review computational approaches that are of at least some relevance for chemical optimization (except standard QSAR techniques). Second, we discuss key methodological features of DeepCOMO. Third, exemplary applications are presented.

## Computational approaches supporting compound optimization

Methods specifically developed to aid in different stages of LO are rare. Approaches that have been adopted and applied in the broader context of LO include statistical multi-parameter balancing and optimization of compound sets to suggest candidates for synthesis [7]. Furthermore, statistical attrition analysis of candidate compounds has also been reported to monitor whether compounds synthesized during LO meet pre-defined quality criteria [8]. Other approaches are focused on computational estimation of physicochemical properties [9], taking into consideration the widely applied rule-based oral availability paradigm [10] or ligand efficiency metrics [11]. Attempts have also been made to parameterize drug-likeness as a desirability function, aiming to generate preferred candidates [12]. Furthermore, computational approaches have been devised to elucidate SAR trends in evolving compound data sets [13] and analyze such trends in a qualitative [14] and quantitative [15] manner. Another interesting methodology that is based upon a statistical framework aims to quantify and visualize LO progression and assess the efficiency and tractability of different projects [16]. In addition, computational tools have been introduced to assess synthetic feasibility of candidate compound [17]. Given that compound design is of major importance during LO, computational approaches providing guidance for compound synthesis have been applied for experimental design [18]. Recently, artificial intelligence has entered the de novo design arena providing complex generative deep learning architectures that are also employed in support of LO campaigns [19, 20].

Taken together, most of the computational approaches that can be considered in the context of LO focus on compound property analysis, candidate selection, or design. By contrast, only very few methods have been introduced to monitor compound optimization and/or SAR trends in different ways [13, 16]. Hence, from this viewpoint, the diagnostic COMO framework was conceptualized to fill a void. As it has further evolved, a unique feature of the approach has become that it bridges between assessing progress in the optimization of ASs and compound design, as exemplified by the DeepCOMO extension introduced in the following.

## Methodology: from COMO to DeepCOMO

### Main principles and diagnostic scoring

COMO combines different scoring schemes including chemical saturation, multi-property, SAR progression, and SAR heterogeneity scores [3–6]. For AS diagnostics, the chemical saturation (S score) and SAR progression (P score) are primary measures for assigning ASs to different development stages. For the calculation of these diagnostic scores, VA populations play a central role because they serve as a representative sample of series-centric chemical space. In addition, the scoring scheme relies on the application of a chemical neighborhood (NBH) principle. Specifically, for chemical saturation and SAR progression diagnostics, the NBH of each existing analog (EA) comprising a series is defined and other compounds falling into the NBH are identified. Accordingly, for a given series, EAs and random samples of a chosen VA population are projected together into a user-defined chemical reference space (typically a vector space formed by numerical chemical descriptors) and the NBH of EAs is defined based on distance relationships between VAs, which determine chemical space coverage, given their large number compared to EAs. In a subsequent step, the proportion of VAs located in NBHs of EAs is calculated, giving rise to the coverage (C) and density (D) scores. The C score quantifies how extensively EAs cover series-relevant chemical space and is defined as:

1 $$C= \frac{V{A}_{NBH}}{V{A}_{all}}$$

where $V{A}_{NBH}$ is the number of VAs falling into any NBH of EAs and $V{A}_{all}$ is the number of all projected VAs.

In addition, the D determines how densely EAs map chemical space by quantifying the overlap of their NBHs:

2 $$D=1-\frac{1}{{d}_{mean}}$$

The term $${d}_{mean}$$ is defined as the number of overlapping NBHs containing VAs ($$NB{H}_{O\_VA}$$) relative to the total number of VAs ($${n}_{NBH})$$ contained in NBHs of EAs:

3 $${d}_{mean}= \frac{NB{H}_{O\_VA} }{{n}_{NBH}}$$

Both scores are complementary in their nature and can be summarized into the S score which is a composite metric defined as the harmonic mean of C and D:

4 $$S= \frac{2CD}{C+D}$$

While the C, D, and S scores are solely devised to quantify chemical saturation, the P score measures the degree of SAR progression as a function of SAR discontinuity in overlapping NBHs of EAs. Hence, for VAs located in overlapping NBHs, the mean pairwise potency range among EAs associated with those NBHs is quantified as the NBH-specific term ${\stackrel{-}{\Delta }}_{i}$:

5 $${\stackrel{-}{\Delta }}_{i}=\frac{2}{{m}_{i}({m}_{i}-1)}\sum_{\begin{array}{c}j,k=1\\ j<k\end{array}}^{{m}_{i}}\left|{\text{pot}}\text{j } \, -{\text{pot}}{\text{k}}\right|$$

Here, ${m}_{i}$ is the number of EAs with overlapping NBHs containing VA(s), ${\text{pot}}{\text{j}}$ and ${\text{pot}}{\text{k}}$ represent the logarithmic (log) potency of EA $j$ and $k$, respectively. The P score for the entire AS represents the mean over ${\stackrel{-}{\Delta }}_{i}$ for all $n$ VAs in overlapping NBHs of EAs applying a weighting scheme ${w}_{i}=\frac{1}{{m}_{i}}$ if ${m}_{i}>1$ and ${w}_{i}=0$ if ${m}_{i}=1$:

6 $$P=\frac{1}{\sum_{i=1}^{n}{w}_{i}}\sum_{i=1}^{n}{w}_{i}{\stackrel{-}{\Delta }}_{i}$$

Hence, large potency variations between structurally similar EAs with overlapping NBHs containing VAs correspond to a strong SAR response to small chemical modifications and, accordingly, to high SAR progression within an AS.

As designed, these scores are robust and practically insensitive to the number of VAs that are used, provided VAs outnumber EAs by at least two to three times [2–4]. Furthermore, the choice of chemical reference spaces for compound distance calculations is variable and can be modified according to the characteristic features and requirements of specific optimization efforts. Herein, a seven-dimensional chemical reference space composed of seven LO-relevant physicochemical descriptors (calculated with RDKit [21]) was used, which was shown to provide sufficient chemical resolution for the characterization of ASs in our previous studies [3–5]. Since the NBH concept plays a central role in the COMO approach, score calculations depend on the definition of a suitable NBH radius that adequately mirrors distances between EAs and VAs. Therefore, this hyper-parameter can be fine-tuned according to different VA populations and/or chemical space representations that might be used [2–4].

### Virtual analog design strategies

**Fig. 1:**
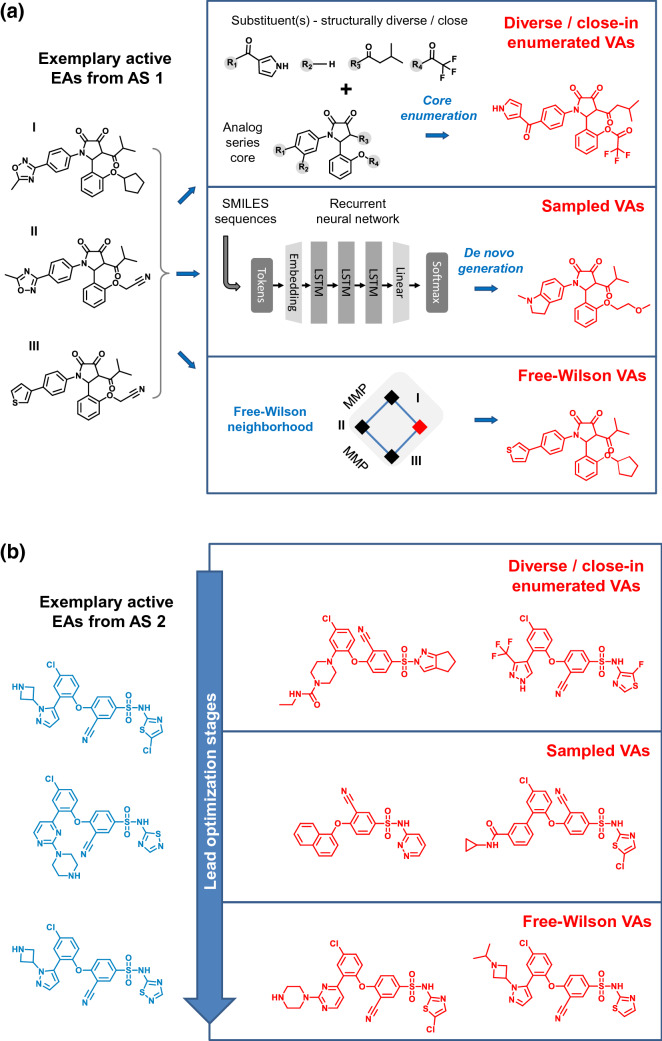
Exemplary analogs and design strategies. **a** On the left, three exemplary EAs (black) from AS 1 are displayed (compounds I, II, and III). Sections on the right illustrate different VA design strategies of DeepCOMO, as discussed in the text. For each strategy, exemplary VAs are shown (red). **b** On the left, three EAs from AS 2 are shown (blue). On the right, exemplary VAs (red) are depicted resulting from the different design strategies according to (**a**)

Different strategies were designed and implemented to generate VA populations as diagnostic tools for COMO scoring and as candidate compounds for optimization efforts [2–4]. These analog design strategies are tailored towards different stages of the LO process (Fig. 1). First, VAs can be generated following a scaffold enumeration procedure. In this case, all substitution sites on the AS core scaffold are decorated with randomly selected terminal fragments according to pre-defined synthetic reactions. For ASs with multiple substitution sites, this procedure can often produce very large and complex VA structures that may not adequately represent AS-specific chemical space. This problem is circumvented by restricting VA size ranges to those of EAs and by randomly decorating one or more substitution sites with a hydrogen atom instead of an organic substituent based on an AS-specific substitution probability [4].

Applying the scaffold-based enumeration approach, two populations of VAs can be generated, termed *diverse* and *close-in VAs*, which differ only in the choice of substituents for enumeration. For diverse VAs, an external pool of R-groups is chosen that have not been used for EAs. For example, such a pool can be extracted from databases of known bioactive compounds. Conversely, for close-in VAs, only substituents obtained from fragmentation of the EAs comprising the AS under study are used for enumeration. Thus, in the case of diverse VAs novel, new chemistry might be introduced, which is more likely to be pursued during early stages of LO. On the other hand, close-in VAs are by design chemically more conservative and should thus be more relevant for mid-stages of LO projects.

In addition to AS scaffold-based enumeration, the Free-Wilson (FW) additivity principle [22, 23] has been adapted and converted into a design strategy for generating VA candidates for late LO stages [5]. Therefore, matched molecular pairs (MMPs) [24] are calculated for EAs of an AS and systematically organized in MMP networks (generated with the NetworkX Python library [25]). Then, analog sets are identified to which FW analysis of substituent contributions is applicable [22]. Such compounds can either be found among EAs (termed *FW EAs*) or they may represent VAs (*FW VAs*) with as of yet unexplored combinations of substituents. FW VAs are designed to become FW prediction targets on the basis of qualifying EAs. By definition, FW VAs can be viewed as a subset of close-in VAs since they contain only R-groups present in the AS. The FW VA population has the advantage of being specifically tailored towards FW potency predictions. Ensuing compound quartets meeting FW requirements consist of three EAs and an FW EA whose putative potency is predicted based upon FW principles. Such quartets represent local mini-QSAR models that have been shown to be surprisingly accurate in many cases and capable of complementing global QSAR strategies for VA prediction and prioritization [5]. Accuracy of FW predictions intrinsically depends on the presence of SAR continuity.

Herein we introduce a strategy for de novo design of VAs (termed *sampled VAs*) using an RNN architecture. This extension of AS-based VA design was inspired by the potential to further extend VA generation by taking information from related compound series or sets into account. Among the many recently introduced approaches for de novo compound design using deep learning, we have given preference to transfer learning (TL) considering the characteristics of the COMO framework.

For COMO-based design, TL [26] is applied to focus a generalized pre-trained generative model by fine-tuning using all EAs of a given AS. The implementation is based upon freely accessible code from the REINVENT 2.0 project [27] as implemented in PyTorch [28], which provides a robust pre-trained generative model (so-called Prior). The model has been trained on more than 1.4 million compounds from ChEMBL (release 25) [29] using tokenized SMILES strings with maximal sequence length of 256 elements [30]. Randomization of SMILES strings was applied as data augmentation technique [30]. As reported, the RNN architecture consists of an embedding layer of size 512, followed by three Long-Short-Term Memory (LSTM) layers of size 512, no drop-out layers, and a linear transformation layer of size 31 (equal to the vocabulary size of the corresponding training data), followed by a softmax function to convert the output into a token probability distribution. Furthermore, adaptive learning rate based on exponential learning rate decay with fixed patience was used [30] and the ADAM optimizer was applied [31]. In addition, a custom Uniformity-Completeness Jensen-Shannon Divergence (UC-JSD) metric [32] was used for estimating model performance. Further details are provided in the source publications [27, 30]. Since typically more than 99% of the compounds sampled using the Prior model have valid SMILES syntax [30] this model can serve as a starting point for TL on the basis of small and structurally confined sets of compounds such as ASs. During multiple epochs, the Prior model is fine-tuned to focus on AS-specific chemical space and generate complementary VAs. As introduced herein, DeepCOMO represents the TL-based extension of COMO’s analog design capacity.

### Potency prediction

To prioritize VAs for synthesis, potency prediction approaches are applied. In practice, it is hardly possible to systematically generate reliable linear or non-linear machine learning regression models for given ASs [5]. This is often due to their confined size, which limits the applicability of machine learning, and also to the presence of series-specific chemical features and SAR discontinuity in AS, both of which might constrain predictive modeling. Furthermore, regression models predict potency values for all VAs, which is also an approximation at best since VAs might often be inactive. However, compounds predicted to be most potent within VA populations principally represent preferred candidates for further consideration. For large ASs, we generally attempt to build global support vector machine regression (SVR) [33] and linear ridge regression [34] models to prioritize VAs. In addition, for all ASs, local FW predictions are attempted, which are supported by the generation of FW VAs for a given AS [5, 6], as described above.

## Exemplary applications

To illustrate the different stages of DeepCOMO analysis, two exemplary ASs were selected as model series mimicking practical LO applications. These two ASs were obtained from our in-house high-confidence activity data version of ChEMBL (release 26) [29]. From this compound database, ASs were extracted using the compound-core-relationship algorithm [35]. Initially, all active compounds were subjected to systematic fragmentation of acyclic single bonds. Subsequently, resulting compound cores were organized into different series. To ensure that algorithmically generated AS cores contained synthetically accessible substitution sites, compound fragmentation was guided by 12 retrosynthetic rules [36, 37] and augmented by nine additional synthetic reactions [38] implemented with the aid of the OpenEye cheminformatics toolkit [39].

### Selected analog series

The ASs studied here (termed AS 1 and AS 2) were active against the P2X purinoreceptor 3 (AS 1) and the sodium channel protein type IX alpha subunit (AS 2) and consisted of 219 and 158 analogs, respectively. For all compounds, IC_50_ measurements were available and recorded as negative logarithmic potency values (pIC_50_). The composition of AS 1 and 2 is summarized in Table 1. These ASs were selected for several reasons. They were among the largest ASs that we algorithmically extracted from public domain data. Furthermore, these ASs were of moderate structural complexity and contained different core structures with four (AS 1) and three (AS 2) substitution sites, hence providing ample opportunities for analog design. Figure 1a and b shows exemplary analogs from AS 1 and 2, respectively.

**Table 1:** Analog series characteristics

Analog series ID	1	2
Biological target	P2X purinoreceptor 3	Sodium channel protein type IX alpha subunit
ChEMBL target ID	2998	4296
Potency measurement type	IC50	IC50
# EAs	219	158
# EAs in Free-Wilson NBHs	183 (84%)	45 (28%)
# substitution sites	4	3
# unique substituents	70	133
Analog series core		
C score	0.43 (± 0.01)	0.18 (± 0.02)
D score	0.90 (± 0.00)	0.73 (± 0.03)
S score	0.58 (± 0.01)	0.29 (± 0.02)
	0.55 (± 0.03)	0.95 (± 0.05)

*EA* existing analog, *NBH* neighborhood

### Diverse, close-in, and Free Wilson virtual analogs

Alternative analog design strategies are schematically illustrated in Fig. 1a. As discussed in detail below, TL produced initial sets of 51,200 SMILES representations per AS. For comparison, equally sized sets of diverse and close-in analogs were generated utilizing all substitution sites per AS. Diverse VAs were randomly enumerated using a pool of 44,636 substituent fragments comprising at most 13 atoms that were extracted from bioactive compounds in ChEMBL (release 26). For enumerating close-in VAs, series-based sets of 70 (AS 1) and 133 substituents (AS 2) were used. Different from diverse and close-in VAs, the number of FW VAs per AS is not variable but depends on intra-series structural relationships, the corresponding distribution of MMPs, and the potential to complement FW NBHs formed by EAs with FW VAs (see above). For AS 1 and 2, a total number of 907 and 3167 FW VAs was obtained, respectively. Figure 1a and b show exemplary VAs for AS 1 and 2, respectively.

### Diagnostic scoring

Next, chemical saturation and SAR progression scores were calculated for both series using the respective close-in VAs as diagnostic VA populations. Therefore, sets of 1000 VAs were randomly selected for 10 independent score calculations, producing very similar results. Mean scores are reported in Table 1. These scores clearly differentiated between the two ASs. Although AS 1 contained only ~ 25% more compounds than AS 2, it was found to be chemically much more saturated (S score = 0.58) with high substantial coverage of chemical reference space (C score = 0.43) and particularly high density of coverage (D score = 0.90). By contrast, chemical saturation of AS 2 was significantly lower (S score = 0.29), resulting from low coverage (C score = 0.18) of chemical space and more moderate density of coverage (D score = 0.73). However, a different picture emerged when SAR progression scores were compared. Here, AS 2 displayed much stronger SAR responses (P score = 0.95) than AS 1 (P score = 0.55), reflecting the presence of higher SAR discontinuity in overlapping NBHs of EAs. Hence, AS 1 was characterized as a further explored compound series with higher series-specific chemical saturation and more balanced potency variations among analogs. On the other hand, the scores indicated that AS 2 still had significantly potential for obtaining analogs with further improved potency. Thus, on the basis of this comparison, AS 1 was categorized as a later-stage series, whereas AS 2 represented an early-/mid-stage series. Notably, conclusions drawn from scoring were fully consistent with the numbers of FW NBHs and participating EAs detected in both ASs. While the subset of FW EAs from AS 1 amounted to 183 (84%) EAs associated with at least one FW NBH, this was the case for only 45 (28%) of the EAs from AS 2, hence reflecting the more advanced development stage of AS 1. Based on these diagnostic findings, one can then decide which VA design strategy would be preferred to generate additional candidate compounds. For instance, AS 1 is likely to benefit from FW VAs as potential candidates (high structural similarity to EAs), given its advanced development stage. On the other hand, for AS 2, a more explorative design strategy would be preferred to further diversify candidate compounds.

### Transfer learning

The TL extension included in DeepCOMO was then applied to sample different VAs, aiming to navigate from generalized drug-like space towards narrowly confined series-centric space and further extend VA design.

**Fig. 2:**
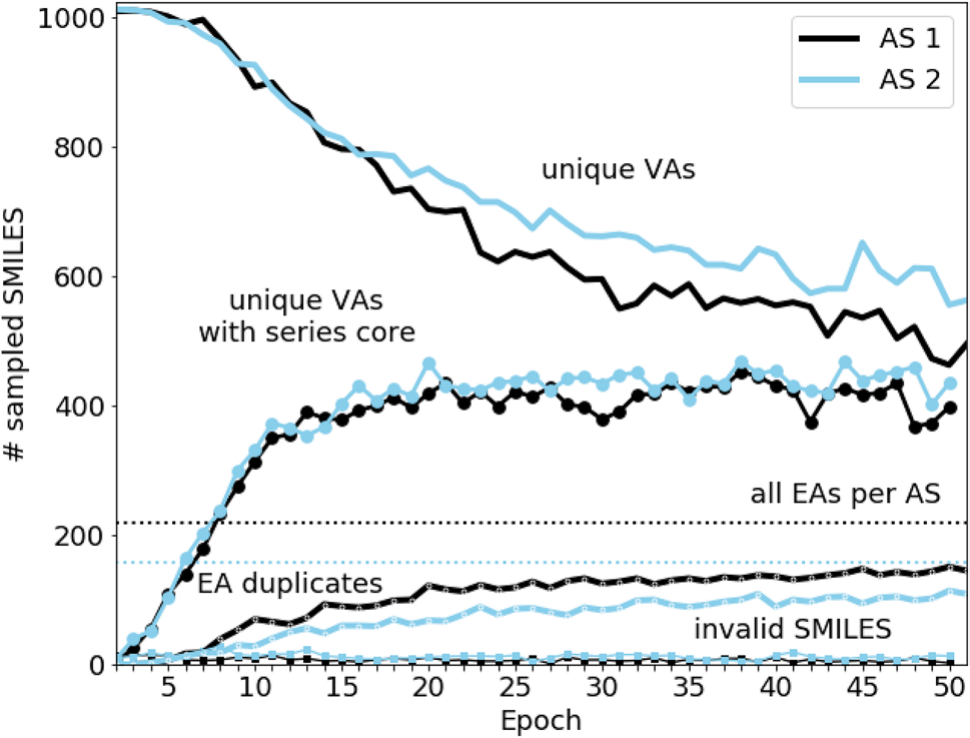
Design of virtual analogs via transfer learning. Shown is the evolution of multiple parameters across 50 epochs of sampling VAs via transfer learning for AS 1 (black lines) and 2 (blue). The x-axis reports the number of epochs and the y-axis the number of sampled VAs (SMILES strings). Curves with filled circles monitor increasing numbers of sampled VAs containing their AS cores. Dotted horizontal lines indicate the number of EAs for each AS. Curves below these lines record the number of duplicated (reproduced) EAs of each AS. At the bottom, curves with squares monitor the fraction of sampled VAs with invalid SMILES strings

The generative model was trained for 50 epochs with 1024 sampled VAs per epoch obtained as SMILES strings, which resulted in a total of 51,200 initially sampled strings per AS. Then, the population of sampled VAs was analyzed with respect to model TL performance. Because TL was increasingly focused on a specific AS core structure a well-performing model should be capable of generating many chemically meaningful structures and unique compounds similar to yet chemically distinct from EAs. Figure 2 shows the evolution of the TL model during training and fine-tuning. Beginning with epoch 1, the generalized Prior model produced a uniform random VA sample without compounds containing the AS cores. However, over the course of only few epochs, the model rapidly learned to sample increasing numbers of compounds similar to EAs, as indicated by the steep rise of the curves accounting for the proportion of sampled VAs with AS cores. The models also reproduced EAs from the training sets (Fig. 2), confirming focused sampling of VAs. Furthermore, the apparent focusing effect was accompanied by a similarly steep decrease in the total numbers of unique sampled VAs. By the 50th epoch, less than 50% and 60% of the sampled VAs represented unique compounds for AS 1 and 2, respectively. Around the 30th epoch, the proportion of generated VAs sharing the AS cores or reproduced EAs reached a plateau at which the ratios between the different curves remain relatively constant. By the 50th epoch, approximately 67% of the EAs of both ASs were reproduced within a single epoch run, whereas the fraction of unique sampled VAs containing the AS core was consistently above 80% and 75% for AS 1 and 2, respectively. Taken together, the analysis revealed successful focusing of the TL model for both ASs, with increasing levels of redundancy when sampling VAs.

**Fig. 3:**
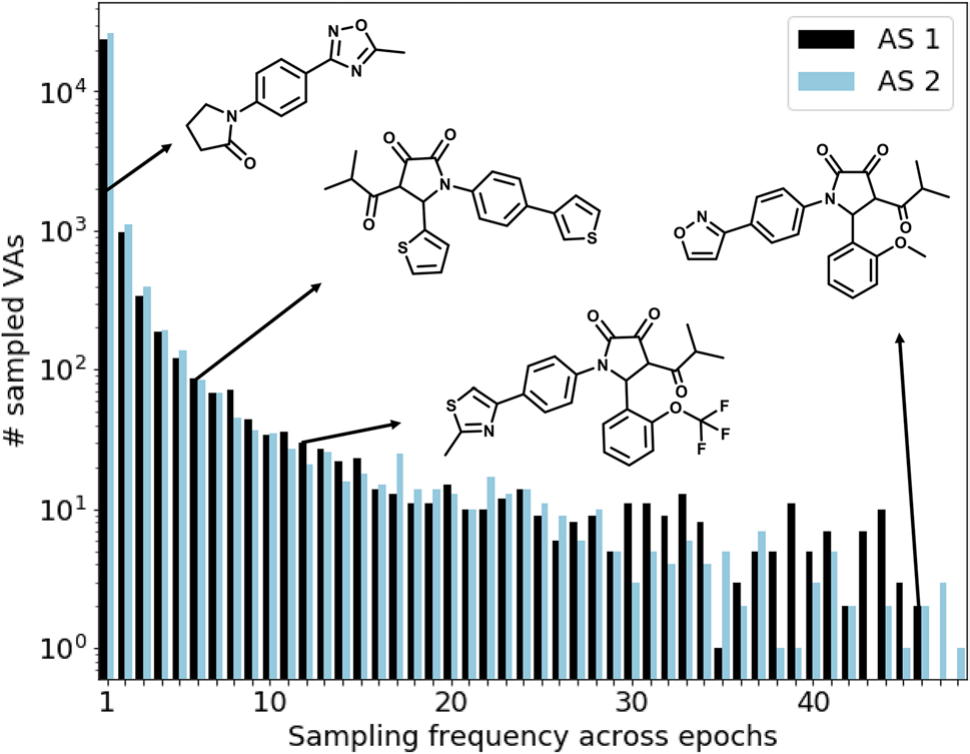
Sampling frequencies of virtual analogs. The bar plot reports the frequency of occurrence for sampled VAs during TL (AS 1, black; AS 2, blue). The x-axis reports the number of epochs and the y-axis the numbers of VAs falling into each category on a logarithmic (log) scale. For AS 1, exemplary sampled VAs with different sampling frequencies (indicated by the black arrows) are depicted

Next, we analyzed how effectively the TL model sampled VAs across different epochs. The 50 training epochs with a SMILES sample size of 1024 produced a total of 26,081 and 28,592 unique VA structures for AS 1 and AS 2, respectively, which corresponded to ~ 51% and ~ 55% of all sampled SMILES strings for AS 1 and AS 2, respectively. These ratios were a consequence of increasing sampling of duplicate structures and reproduced EAs within individual epochs (Fig. 2). Since this effect propagated throughout the fine-tuning phase, some VAs were sampled in multiple epochs, whereas others were obtained in very few or just one. As illustrated in Fig. 3, the majority of sampled VAs was generated during only one of the epochs. In addition, the number of VAs sampled in multiple epochs significantly decreased over increasing number of epochs. In Fig. 3, four exemplary structures of VAs of AS 1 are depicted that were sampled in different numbers of epochs. These VAs were selected from the batch generated during the 40th epoch when the output of the generative model was stable (Fig. 2). The VA in the upper left corner in Fig. 3 was sampled only once during the 50 generative epochs because it did not contain core of AS 1 but represented a simpler structure, consistent with the initial generalization capability of the Prior model. The next sampled VA to the right contained a substructure of the AS 1 core.

In which the signature *o*-alkoxyphenyl ring at the gamma lactam position was substituted with a thiophene ring. Although this sampled VA not contain the entire AS 1 core, it was sampled six times (in epochs 8, 16, 26, 40, 41, and 48) including the late stages of TL. Thus, the model consistently diversified structural features of sampled VAs including core modifications, even after focusing on the same core over many epochs. These observations mirrored an intrinsic advantage of the deep generative architecture over the simpler VA enumeration strategies based upon a conserved AS core. The third VA from the left in Fig. 3 contained the complete core of AS 1, but was only sampled during 12 of 50 epochs. This is likely due to the varying frequency of occurrence of individual substituents among the EAs used for training. For example, the *o*-methylthiazole and trifluoromethyl groups were only present in two and 15 training instances, respectively. By contrast, the VA on the right with different more frequently occurring substituents was most frequently sampled in 46 epochs. These comparisons illustrate the spectrum of structural modifications of sampled VAs obtained by AS-centric fine-tuning of the model, yielding an expansion of VA space.

### Comparison of virtual analog populations

**Fig. 4:**
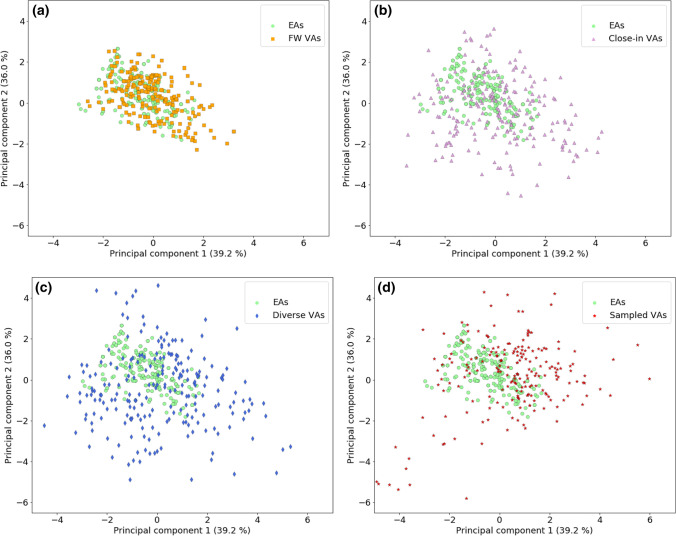
Chemical space coverage. In **a**–**d**, PC plots compare the coverage of chemical reference space by AS 1 with its four VA populations. Sampled VAs were randomly selected from the batch of the 40th epoch. For each principal component, it is reported for how much of the original data variance it accounts

Next, the coverage of AS-specific chemical space by VA populations produced using the four design strategies of the DeepCOMO framework was analyzed and compared. First, the overlap between differently designed VA populations (and between VAs and EAs) was determined, as reported in Table 2. From the pools of diverse and close-in VAs of AS 1 and 2, subsets were randomly selected to match the number of sampled VAs. For both ASs, nearly all EAs were reproduced by TL. However, the TL model sampled only 69% of the FW VAs of AS 1 and 45% of AS 2. Apart from this, the overlap between different compound populations was generally larger for AS 2 than AS 1. The largest difference was observed between the overlap of close-in VAs with other compound populations. Nonetheless, in both cases, all four VA design strategies produced significant numbers of unique compounds, indicating their principal complementarity in charting analog space. In the next step, VA distributions in series-centric chemical space were compared. Therefore, EAs and equally sized random samples of all VA populations were projected into the descriptor-based seven-dimensional reference space and subjected to dimension reduction using principal component analysis (PCA). Plots were generated using the first two principal components. For both series, equivalent observations were made. For AS 1, pairwise comparisons of the EA distribution and different VA distributions are shown in Fig. 4a–d. As expected, the FW VA population mapped most closely to EAs (Fig. 4a), consistent with the underlying FW NBH-directed design strategy. Close-in VAs were already more widely distributed but mostly covered regions proximal to EA (4b). For diverse VAs, a more extensive spread was observed (4c). For PCA, sampled VAs shown were exclusively selected from the batch obtained for 40th epoch and thus represented a late-stage “snapshot” of the fine-tuned TL model. Deriving this VA population combined information from compounds with varying structural relationships to EAs and facilitated additional core modifications. Accordingly, the comparison of sampled VAs and EAs in Fig. 4d revealed a combination of different patterns observed for other VAs including strong focusing on subsets of EAs, proximal mapping to many others, but also substantial diversification. Hence, the distribution of sampled VAs combined and further extended characteristics of VA populations obtained with simpler design strategies.

**Table 2:** Virtual analogs statistics

Analog series ID	1	2
# experimental EAs	219	158
# FW VAs	907	3167
# unique sampled VAs # diverse VAs # close-in VAs	26,295	28,748
Sampled VAs & EAs	214	156
Sampled VAs & FW VAs	624	1436
Sampled VAs & close-in VAs	208	1669
Sampled VAs & diverse VAs	0	18
Close-in VAs & diverse VAs	35	37
FW VAs & close-in VAs	53	2909
FW VAs & diverse VAs	0	0

*EA* existing analog, *VA* virtual analog, *FW* Free-Wilson, *&* intersection

### Synthetic accessibility of virtual analogs

**Fig. 5:**
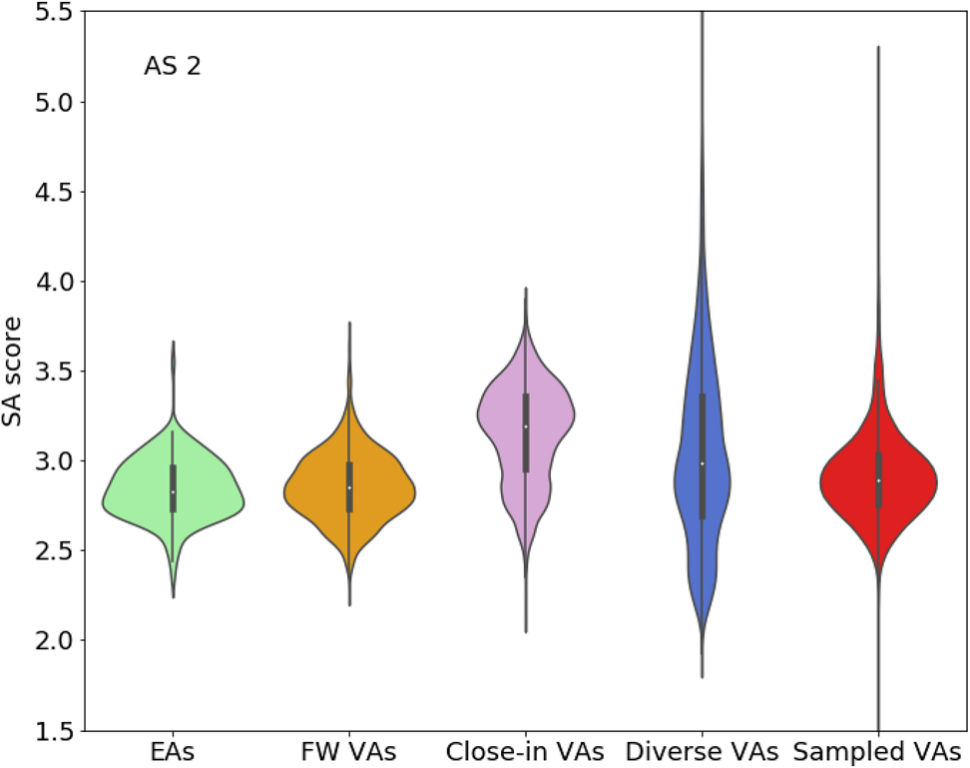
Synthetic accessibility. Violin plots report SA score distributions for AS 2 and its VA populations

Synthetic accessibility of VAs continues to represent a much discussed topic, especially for compounds generated using deep learning architectures. Accordingly, we also calculated and compared synthetic accessibility (SA) scores [17] for our VA populations (using the public RDKit implementation available on GitHub [17]). The SA score ranges from 1 to 10 and accounts for fragment contributions to compounds based upon empirical assessment of synthetic building blocks, stereo chemistry, and non-standard structural features [17]. Increasing scores indicate the presence of chemically complex compounds that are increasingly challenging to synthesize. As shown in Fig. 5, VA populations for AS 2 yielded SA scores that were comparable to or only slightly higher than EA scores, hence indicating general synthetic feasibility. Equivalent observations were made for AS 1. Overall broadest score distributions including subset of higher scoring compounds were observed for diverse VA, which one might expect, as these VAs combine substituent fragments from the entire universe of current bioactive compounds, regardless of their core structures.

### Prioritization of virtual analogs

**Fig. 6:**
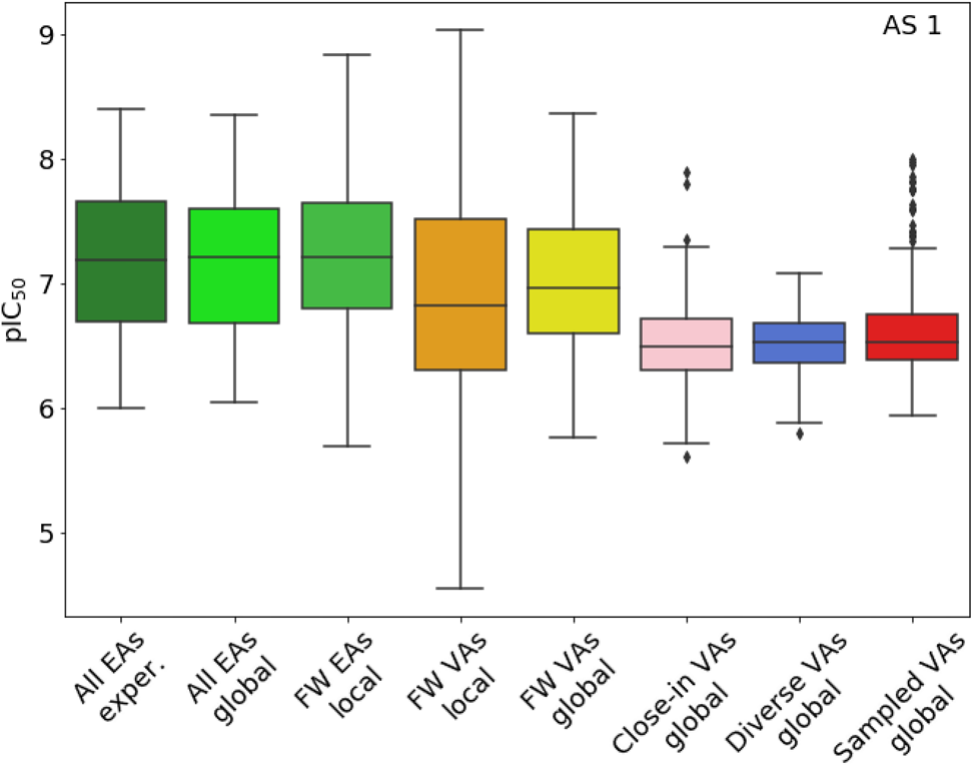
Compound potency predictions. Box plots report potency predictions for AS 1 and its VA populations using global (SVR) and local (FW) models. The latter models are only applicable to FW EAs and FW VAs

VAs predicted to be most potent represent preferred candidates for further optimization efforts. For AS 1 and AS 2, global series-based and local FW NBH-based prediction models were derived. For global predictions, SVR [33] models were trained via three-fold double cross-validation [40]. For model building, a folded (2048-bit) version of the extended connectivity fingerprint with bond diameter of 4 (ECFP4) [41] was used in combination with the Tanimoto kernel [42] as a similarity function. All calculations were carried out using Python’s scikit-learn library [43]. For training, 517 (AS 1) and 1135 (AS 2) compounds with activity against each AS target were collected from ChEMBL that did not belong to the AS (representing structurally diverse active compounds) and combined with 50% of the respective AS. The remaining 50% of the EAs were used as an external validation set. The SVR models were then used to predict the potency of these EAs and of the different VA populations. Furthermore, FW NBH-based potency predictions were carried out for FW VAs and qualifying FW EAs. For AS 1, prediction results are reported in Fig. 6 (comparable observations were made for AS 2). Accurate retrospective potency predictions were obtained for EAs using both local and global models, with R^2^ values of 0.84 (± 0.0) and 0.81 (± 0.03), respectively, and mean absolute errors of 0.18 (± 0.0) and 0.2 (± 0.03), respectively. For VA populations, global models generally predicted lower potency values than for EAs, as observed previously [5]. Overall highest potency was predicted using local and global models for FW VAs, which most closely resembled EAs. However, for all except diverse VAs, at least few “outlier” compounds were predicted to have higher potency than most of the EAs. These compounds provide focal points for VA prioritization as potential candidates depending on the development stages of an AS, as assessed by COMO scoring.

## Conclusions

In medicinal chemistry, LO is still more of an art form than a scientific exercise following firm and generally applicable rules. It is governed by the recurrent need to decide which compounds to make next. These largely experience- and chemical intuition- driven optimization efforts greatly benefit from any approaches that are capable to rationalize at least a part of the proceedings and provide decision support beyond subjective judgment. In principle, computational methods are prime candidates to support LO. However, as discussed herein, only few relevant approaches besides standard QSAR techniques have thus far been introduced to aid in this process. Within this scientific context, COMO was conceptualized, originally as a diagnostic framework, and then further expanded to bridge between chemical/SAR analysis and compound design. Herein, we have discussed key features of the methodology and presented the DeepCOMO extension for further advanced compound design. DeepCOMO provides four design strategies that yield complementary VA populations with varying AS-centric chemical space coverage. It has been applied to two exemplary ASs at different development stages, illustrating the spectrum of its diagnostic and design components and rationalizing how to combine these components for COMO-guided decision making. We hope that our discussion and findings presented herein might catalyze the development of additional computational concepts and methods to aid in compound optimization efforts, which would certainly be beneficial for the practice of medicinal chemistry. Applications of DeepCOMO in practical medicinal projects are underway.
